# Computational Analysis of Transcriptional Circuitries in Human Embryonic Stem Cells Reveals Multiple and Independent Networks

**DOI:** 10.1155/2014/725780

**Published:** 2014-01-09

**Authors:** Xiaosheng Wang, Chittibabu Guda

**Affiliations:** ^1^Department of Genetics, Cell Biology and Anatomy, University of Nebraska Medical Center, Omaha, NE 68198-5805, USA; ^2^Bioinformatics and Systems Biology Core, University of Nebraska Medical Center, Omaha, NE 68198-5805, USA

## Abstract

It has been known that three core transcription factors (TFs), NANOG, OCT4, and SOX2, collaborate to form a transcriptional circuitry to regulate pluripotency and self-renewal of human embryonic stem (ES) cells. Similarly, MYC also plays an important role in regulating pluripotency and self-renewal of human ES cells. However, the precise mechanism by which the transcriptional regulatory networks control the activity of ES cells remains unclear. In this study, we reanalyzed an extended core network, which includes the set of genes that are cobound by the three core TFs and additional TFs that also bind to these cobound genes. Our results show that beyond the core transcriptional network, additional transcriptional networks are potentially important in the regulation of the fate of human ES cells. Several gene families that encode TFs play a key role in the transcriptional circuitry of ES cells. We also demonstrate that MYC acts independently of the core module in the regulation of the fate of human ES cells, consistent with the established argument. We find that TP53 is a key connecting molecule between the core-centered and MYC-centered modules. This study provides additional insights into the underlying regulatory mechanisms involved in the fate determination of human ES cells.

## 1. Introduction

Pluripotency and self-renewal are two defining properties of embryonic stem (ES) cells. Pluripotency is the capacity to generate all cell types, while self-renewal is the capacity to maintain ES cells in a proliferative state for prolonged periods [1]. It has been of great interest to know how the ES cells balance the two statuses of pluripotency and self-renewal. It has been found that the three core transcription factors (TFs) NANOG, OCT4, and SOX2 collaborate to regulate pluripotency and self-renewal of human ES cells in the form of a regulatory circuitry [2]. NANOG is a gene expressed in ES cells, which plays a key role in maintaining the pluripotency of ES cells. Downregulation of NANOG will result in differentiation, while expression will block differentiation of ES cells. OCT4, also known as POU5F1, is a gene encoding the protein that is critically involved in the self-renewal of undifferentiated ES cells. OCT4 expression level must be within a certain range to maintain the undifferentiated status of ES cells. SOX2 gene encodes a member of the SRY-related HMG-box (SOX) family of TFs involved in the regulation of embryonic development and in the determination of cell fate. It plays a critical role in the maintenance of embryonic and neural stem cells. SOX2 has been shown to interact with PAX6 [3], NPM1 [4], and OCT4 [5] and cooperatively regulate REX1 with OCT3/4 [6].

Boyer et al. have identified the bound genes of the three core TFs *in vivo* by genome-scale location analysis [2]. They found that OCT4 is associated with 623 (3%) promoter regions of the known protein-coding genes in human ES cells, while SOX2 and NANOG are associated with 1271 (7%) and 1687 (9%) genes, respectively. Further, they identified a set of 353 genes (Table S1; see Supplementary Material available online at http://dx.doi.org/10.1155/2014/725780) that are cobound by all the three TFs in human ES cells and found that this set includes a substantial number of genes that encode homeodomain TFs, which are important in developmental regulation of ES cells. These discoveries suggested that the three TFs function together to control pluripotency and self-renewal of human ES cells. Hereafter, we refer to the set of 353 genes as the core-bound genes.

MYC is another important transcriptional regulator in ES cells, which is involved in somatic cell reprogramming and cancer [7]. Takahashi and Yamanaka generated induced pluripotent stem cells (iPSCs) by forced expression of four transcriptional factors (OCT3/4, SOX2, KLF4, and MYC) in mouse embryonic and adult fibroblast cultures [8] and later in adult human dermal fibroblasts [9]. These studies indicate that MYC also plays a key role in controlling pluripotency and self-renewal of ES cells, although it may act in a distinct way from the core module [1, 7, 10]. However, the precise mechanism by which the transcriptional regulatory networks control the activity of ES cells remains unclear. It is likely that the transcriptional circuitry of ES cells is regulated by multiple core TFs using independent networks, to regulate self-renewal and differentiation of human ES cells.

In this study, we reanalyzed the core-bound genes using Ingenuity Pathway Analysis tool (IPA, Ingenuity Systems, http://www.ingenuity.com/) and the gene set enrichment analysis (GSEA) software [11]. Important networks, biological functions, and pathways associated with the gene sets were annotated. We induced the TFs that bind to the subsets of the core-bound genes with DAVID tool [12, 13] and analyzed the transcriptional network based on the induced TFs. In addition, we compared the regulatory targets of MYC with the core-bound genes and also the MYC-centered and core-centered regulatory modules to determine if these regulatory circuits operate independently or collaboratively.

## 2. Materials and Methods

We obtained the set of 353 genes that are cobound by NANOG, OCT4, and SOX2 in human ES cells from Boyer et al. [2]. We downloaded the 189 TFs which have been experimentally verified to contribute to transcriptional regulation in human ES cells from the literature [14]. The MYC targeted gene lists in human ES cells were obtained from the literature [15]. The gene lists for the core module and the MYC module in ES cells were downloaded from Kim et al. [7].

We inferred significant networks, biological functions, and pathways associated with gene sets using the core analysis tool in IPA (Ingenuity Systems, http://www.ingenuity.com/). IPA is a system that yields a set of networks relevant to a list of genes based on the preserved records contained in the Ingenuity Pathways Knowledge Base (IPKB). For the input of a gene set into IPA, its core analysis tool will map the gene list to the IPKB and then algorithmically generate molecular networks, biological functions, and canonical pathways that are most likely relevant to the input gene list. IPA is the primary tool used by us to produce visualized gene regulatory networks for analysis of transcriptional regulatory circuits in human ES cells.

We classified genes into different gene families using the “Investigate Gene Sets Tool” in the molecular signatures database (MSigDB) of the gene set enrichment analysis (GSEA) software [11]. We induced the TFs that bind to subsets of a given gene list using the “Functional Annotation Tool” in DAVID [12, 13]. DAVID provides a category called “UCSC_TFBS” in the “Protein_Interactions” option of the functional annotation tool. For an input gene list, DAVID analysis will output a list of TFs that bind subsets of the given gene set. For each identified TF, its binding genes and corresponding *P* values are provided.

## 3. Results and Discussion

### 3.1. Functional Analysis of the Core-Bound Genes

We first classified the core-bound genes into different gene families using the gene set enrichment analysis (GSEA) software [11].  Table 1 shows that a significant proportion of genes are TF genes (90 of 353), suggesting that the core TFs in turn bind and regulate a large number of other TF genes in the ES cells [2]. The genes encoding homeodomain proteins also have a large proportion in the core-bound genes (34 of 353), all of which encode homeodomain TFs. The homeodomain TFs have been shown to play key roles in fate-determination of ES cells by contributing to the core regulatory networks. It should be noted that there are 11 oncogenes in the core-bound genes, which is indicative of certain similarities between ES and cancer cell transcription programs [7, 14].

**Figure 1:**
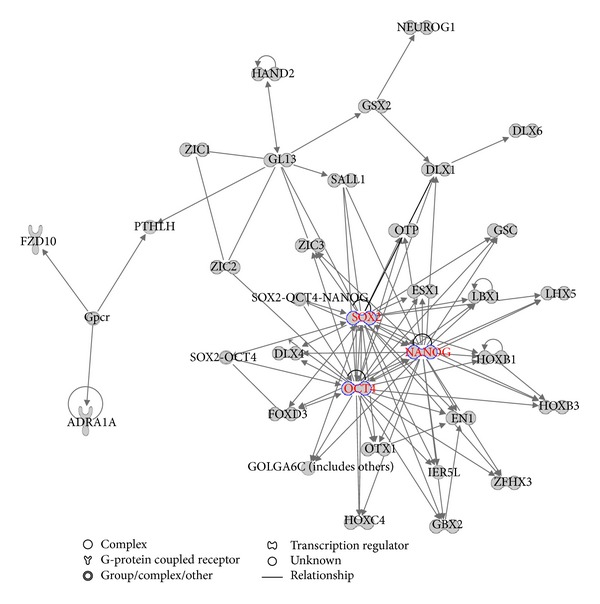
Top network related to the core-bound genes. The three core TFs form hub nodes in the network as highlighted in red color.

Network analysis of the 353 core-bound genes using IPA (Ingenuity Systems, http://www.ingenuity.com/) shows that the top network involves 32 genes among which the three core TFs, NANOG, OCT4, and SOX2, were hub nodes in the network, and formed interconnected autoregulatory and feedforward circuitry (Figure 1). Biological function analysis shows that the core-bound genes are mostly relevant to regulation of gene expression and developmental processes. The developmental processes include nervous system development and function, embryonic development, and organ, organismal, tissue, and cellular development. The six most significant pathways associated with the core-bound genes include transcriptional regulatory network in embryonic stem cells (*P* value *≈* 10^−47^), role of OCT4 in mammalian embryonic stem cell pluripotency (*P* value *≈* 10^−8^), human embryonic stem cell pluripotency (*P* value *≈* 10^−7^), embryonic stem cell differentiation into cardiac lineages (*P* value *≈* 10^−5^), Wnt/*β*-catenin signaling (*P* value *≈* 10^−4^), and role of NANOG in mammalian embryonic stem cell pluripotency (*P* value *≈* 10^−4^). These results corroborate the previous findings that the core TFs and the core TF-bound genes are essential for maintaining the pluripotency of ES cells.

### 3.2. Identification of Other TFs That Target the Core-Bound Genes

**Figure 2:**
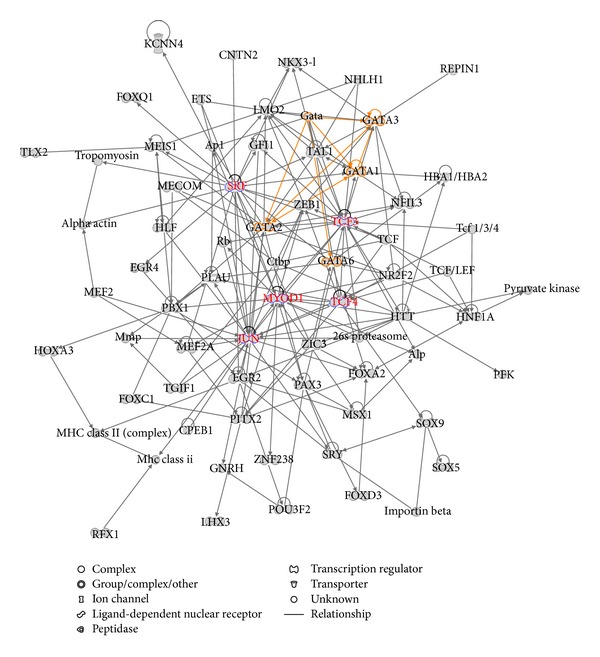
A significant regulatory network related to the 145 computationally predicted human ES cell related gene set. The GATA family of TFs and other important TFs are highlighted.

In addition to the three core TFs, many other TFs also bind to the same set of core-bound genes. Using DAVID tool [12, 13], we identified 145 TFs, where each TF bound at least 30 genes in the core-bound gene set (Table S2). We referred to the 145 TFs as the computationally predicted TFs associated with transcriptional regulation in human ES cells because these TFs are regulating the same genes that are also transcriptionally regulated by the core TFs. We carried out a network analysis for the 145 TFs using IPA. Our goal is to see if these extended TFs are part of the original core TF circuitry or if they use independent circuitries to regulate the core-bound genes.  Figure 2 presents a significant regulatory network related to the 145 TF gene set. The network involved 70 nodes among which the GATA transcription factor family members (GATA1, GATA2, GATA3, and GATA6) form interconnected autoregulatory and feedforward circuitry (in yellow), suggesting that GATA TFs are active in transcriptional regulation in human ES cells. The network also shows that several TF genes such as TCF3, TCF4, SRF, MYOD1, and JUN form hub nodes (in red), suggesting their significance in the same circuitry. Biological function analysis indicated that the TFs were significant in regulation of cell and organ development (Figure S1). Pathway analysis indicated that the TFs were mostly involved in the transcriptional regulatory network in embryonic stem cells pathway (*P* value *≈* 10^−12^) (Figure S2), the same result as that shown in the core-bound gene analysis.

In a recent study [14], we have collected 189 TFs that have been experimentally verified to contribute to transcriptional regulation in human ES cells. We found that there were 41 overlaps between the 189 TFs set and the computationally predicted 145 TF set from DAVID program as shown in Table S3.

### 3.3. Extension of Transcriptional Network in Human ES Cells

Boyer et al. have identified the core transcriptional regulatory network in human ES cells in which the three core TFs collaborate to regulate a substantial number of their target genes [2]. We tried to extend the core transcriptional regulatory network based on the combination of the core-bound genes and the TFs that bind to subsets of the core-bound genes. The combined gene set was composed of the 353 core-bound genes and the aforementioned 145 TF genes. The five most significant networks associated with the combined gene set were summarized in Table S4. Note that 4 of the 5 networks were associated with embryonic development. Below, we describe the regulatory circuits of important TFs or families of TFs in each network.

Figure S3 shows that the three core TFs (NANOG/OCT4/SOX2) act as the hub genes in the regulatory network, which is anticipated; Figure S4 shows that TP53 is the center of the regulatory network with the third highest score, indicating that TP53 plays an active role in the transcriptional circuit for human ES cells. In fact, many experimental lines of evidence have revealed that TP53 plays a key role in determining the fate of ES cells [16–20]. Silencing of the tumor suppressor gene TP53 significantly increased the reprogramming efficiency of human somatic cells [17]. Some studies have shown that the p53 pathway can maintain the homeostasis of self-renewal and differentiation of human ES cells [21–23].

**Figure 3:**
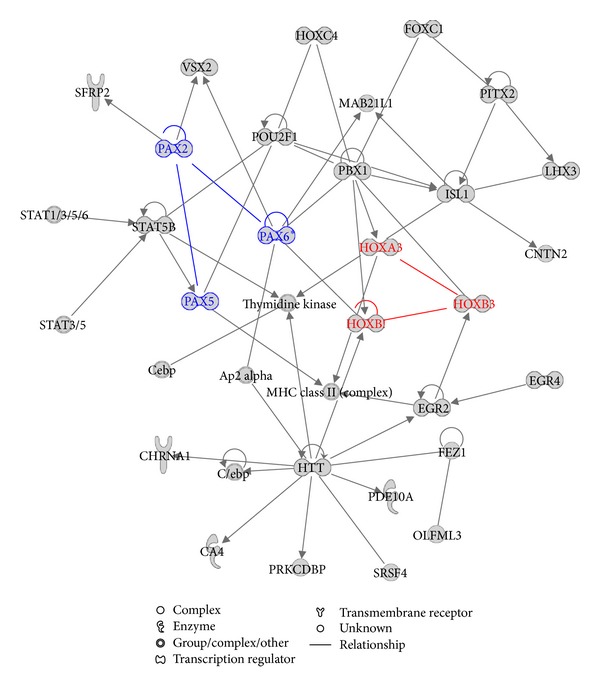
The network related to the combined gene set with the fourth highest score. The combined gene set is made up of the 353 core-bound genes and the 145 computationally predicted human ES cell related TF genes. The HOX and PAX families of TF genes are highlighted in red and blue color, respectively.

Figure 3 shows three important gene families, HOX, PAX, and STAT, that are highly active in the regulatory network. The members of PAX and HOX gene family form autoregulatory loop and also regulate members of other gene families in the network. Interestingly, within individual autoregulatory loop, PAX2 and PAX6 self-regulate and show bidirectional regulation on each other but with contrary effect: PAX2 positively regulated PAX6, while PAX6 has inhibitory effect on PAX2. Based on the regulatory circuitry shown in Figure 3, we infer that HOX, PAX, and STAT gene families play a very important role in controlling the fate of human ES cells by forming a specific regulatory motif. In fact, these three gene families have been experimentally verified to be important in the regulation of developmental processes of human ES cells. HOX genes encode TF proteins which are master regulators of embryonic development [24]. They are important targets of OCT4, SOX2, and NANOG and often transcriptionally inactive when bound by the core regulators to inhibit differentiation. Our results show that except for regulated by the core regulators (Figure 1), HOX family of genes could form their own internal and autoregulatory loop to control the developmental processes of human ES cells. On the other hand, PAX is a family of tissue-specific TFs containing a paired domain and usually with a partial or complete homeodomain. PAX regulates cell proliferation and self-renewal, resistance to apoptosis, migration of embryonic precursor cells, and the coordination of specific differentiation programs during embryonic development. Therefore, PAX plays an essential role in regulation of the pluripotency and self-renewal of human ES cells [25]. Finally, STAT family of TFs regulate cell growth, survival, and differentiation via activation by JAK (Janus kinase). This pathway is critical for regulation of stem cell self-renewal and differentiation [26].

**Figure 4:**
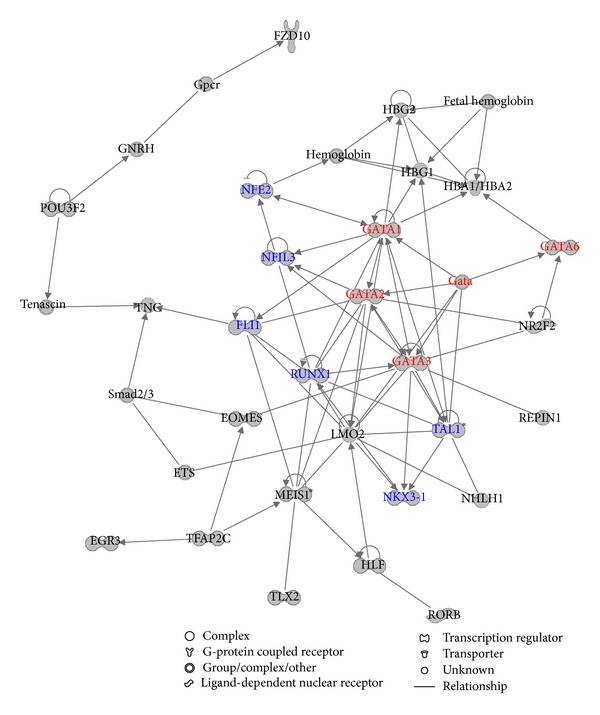
The network related to the combined gene set with the fifth highest score. The combined gene set is made up of the 353 core-bound genes and the 145 computationally predicted human ES cell related TF genes. Red indicates the GATA TF family member and blue indicates the other TFs that interact with the GATA TF family member.

Another network (Figure 4) shows that the GATA family of TFs interconnects and forms regulatory circuit with the other six TFs including NFE2, NFIL3, RUNX1, NKX3-1, TAL1, and FLI1. Therefore we infer that GATA is also important in regulation of pluripotency and self-renewal of human ES cells. Previous studies have revealed that GATA was active in transcriptional regulation in human ES cells through transcriptional coexpression with many other key regulators [25, 27, 28].

Therefore, in addition to the core transcriptional network, we infer that some other transcriptional networks are potentially important in regulation of pluripotency and self-renewal of human ES cells.

Pathway analysis shows that the most significant pathways associated with the combined gene set (353 core-bound genes and 145 TFs) include transcriptional regulatory network in embryonic stem cells (*P* value = 3.73 × 10^−49^), role of OCT4 in mammalian embryonic stem cell pluripotency (*P* value = 4.76 × 10^−11^), Wnt/*β*-catenin signaling (*P* value = 1.08 × 10^−8^), and human embryonic stem cell pluripotency (*P* value = 4.03 × 10^−8^). Apparently, these pathways are strongly associated with the function of regulating the fate of human ES cells.

### 3.4. MYC Transcriptional Network in Human ES Cells

Similar to the core TFs, MYC is a very important TF in the ES cells. A set of 369 genes was identified as MYC targeted genes in human ES cells [26], which are listed in Table S5. We explored the regulatory network involving MYC and MYC target genes using IPA software. As expected, MYC and TP53 turned out as the hub genes in the two most important networks, respectively (Figure S5 and Figure S6). The most significant biological functions associated with this gene set are involved in embryonic, organismal, tissue, and cell development, cell cycle, gene expression, cancer, and so forth. The most significant pathways associated with the gene set included Wnt/*β*-catenin (*P* value = 9.37 × 10^−5^), human ES cell pluripotency (*P* value = 2.14 × 10^−4^), MYC mediated apoptosis signaling role (*P* value = 7.59 × 10^−4^), and so forth. Notably, MYC regulated a cluster of genes that were involved in the human ES cell pluripotency pathway.

**Figure 5:**
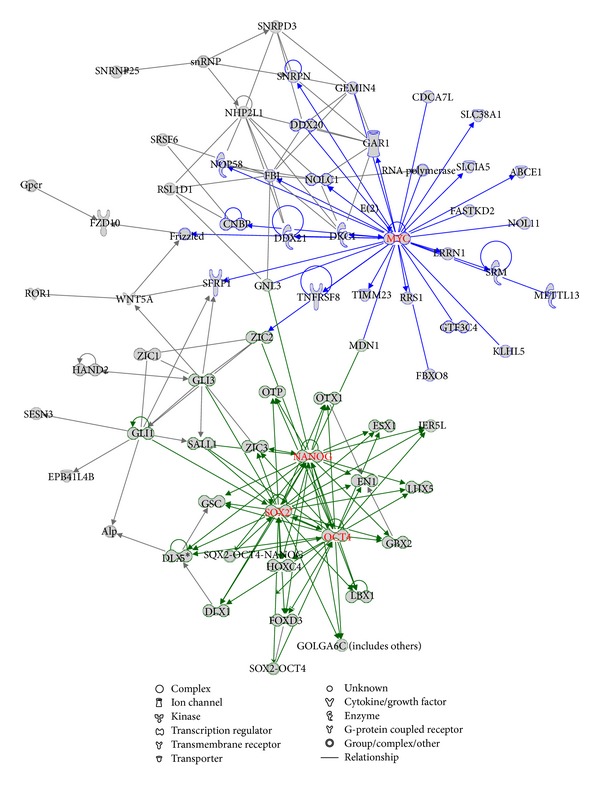
The network based on the combination of the core-bound genes and the MYC targeted genes. The core-centered module and the MYC-centered module are highlighted.

We were also curious to see if MYC and core TFs regulate the same transcriptional circuitry or operate individually. Hence, we carried out a combined network analysis of the core-bound genes and the MYC target genes. There are only 17 overlapping genes between the core-bound gene set and the MYC target gene set that corresponds to only 5% of each target gene set. In fact, the number of overlapping genes between the MYC target gene set and each of the three core TF's target gene sets is also small (50, 19, and 37 for NANOG, OCT4, and SOX2, resp.). The lower overlapping rate supports the previous argument that the MYC-centered regulatory network belonged to a different module from the core transcriptional module in ES cells [1, 7, 10]. Our network analysis clearly shows that there are two separable modules, the core-centered module and the MYC-centered module, which form the transcriptional circuity in the ES cells (Figure 5).

**Figure 6:**
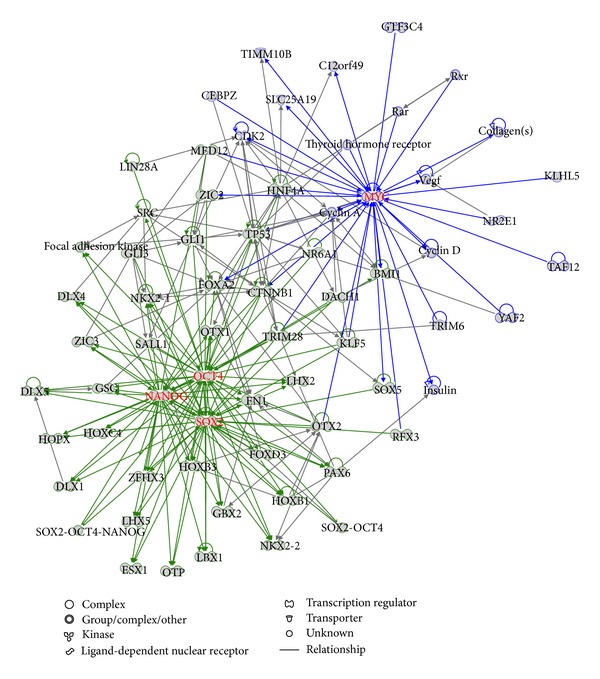
The network based on the combination of the core-bound TF genes and the MYC targeted TF genes. The core-centered module and the MYC-centered module are highlighted.

It has been shown that the core TFs and MYC play key roles in the regulation of ES cells' fate by regulating many TF genes which in turn regulate a large number of other genes [1, 2, 10]. We found that there are 90 TF genes in the 353 core-bound genes and 38 TF genes in the 369 MYC target genes. We carried out an analysis of the regulatory network based on these TF genes only.  Figure 6 shows that the core TFs and MYC form center of the two distinct modules. An interesting finding is that MYC has no connection with any of the three core TFs but interconnects with TP53, which in turn regulates NANOG and is regulated by OCT4. This finding suggests that TP53 has stronger link with the core TFs than MYC and also indicates that TP53 might play a key role in bridging the core-centered and MYC-centered modules.

To further investigate the differences in the regulatory modules of the core- and MYC-centered networks, we obtained two gene sets: a gene set in the core regulatory module and a gene set in the MYC-centered regulatory module, both from the mouse ES cells [7]. We used the human orthologs of the mouse genes in both modules, which contained 75 and 356 genes, respectively (Table S6). There were only three overlapping genes between both modules, again showing that both modules were functionally separate. Similarly, we inferred the significant networks associated with the core module and the MYC module, respectively (Figure S7 and Figure S8). The top 5 pathways associated with both modules were present in Table S7. There are no overlapping pathways between both modules, suggesting that the MYC module and the core module are indeed involved in very different pathway patterns in regulating pluripotency and self-renewal of ES cells.

## 4. Conclusions

It has been found that transcriptional networks were essentially responsible for regulation of pluripotency and self-renewal of human ES cells. Some key TFs like NANOG, OCT4, and SOX2 have been identified to collaboratively control pluripotency and self-renewal by forming interactive regulatory circuits [2, 29]. However, it is presently unclear how the transcriptional networks precisely control the activity of ES cells. It is likely that additional TFs may also regulate the key downstream TFs or form additional regulatory circuits that are involved in the regulation of pluripotency and self-renewal of human ES cells. We have explored an extension of the core transcriptional regulatory network by adding additional TFs into the core transcriptional networks.

Evidence shows that many TFs are involved in both ES cell fate determination and cancerous pathogenesis. For example, oncogene MYC and tumor suppressor gene TP53 have been shown to significantly contribute to the formation of the transcriptional networks that determine the self-renewal or differentiation fate of human ES cells. Several families of human ES cell associated TFs like MYB, E2F, PAX, SMAD, STAT, POU, SP, and GLI are related to cancer [14]. This evidence suggests that ES cell and cancer cells may share essential regulatory mechanisms. Therefore, understanding of how the regulatory network regulates self-renewal or differentiation fate of human ES cells may pave the way for understanding of cancer, and further conquering cancer.

In addition, based on the comparisons of the MYC-centered regulatory module and the core regulatory module in human ES cells, our results suggest that MYC acts independently of the core module in the regulation of pluripotency of human ES cells. In addition, we also showed that TP53 is a key connecting molecule between the core-centered and MYC-centered modules.

Our computational network-based approach supplements the experimental methods to unravel the transcriptional regulatory mechanisms that control pluripotency and self-renewal in the ES cells, although the reliability of our results needs further experimental verification. However, it should be noted that there exist certain limitations in the present methods. First, the information collected by IPA and DAVID databases is from many different studies that are not necessarily specific to human ES cells; hence, the extrapolation of such data to ES cells may lead to false positive information in certain cases. Secondly, as the new findings presented in this study lack experimental verification, it is difficult to assess the sensitivity and specificity of this approach. We plan to collaborate with experimental investigators to validate some of these findings in the future.

## Supplementary Material

Supplementary Tables
**Table S1**: 353 genes that are co-occupied by NANOG, OCT4 and SOX2 in human ES cells.
**Table S2**: 145 genes encoding the TFs that target the core target genes.
**Table S3**: 41 overlapping TFs between the 189 TFs set and the 145 TFs set.
**Table S4**: A summary of the top 5 networks based on the combined gene set which is made up of the 353 core-bound genes and the 145 computationally predicted human ES cell related TF genes.
**Table S5**: MYC target genes in human ES cells.
**Table S6**: Human orthologs of the genes in the core module and the genes in the MYC-centered module in ES cells.
**Table S7**: Top 5 pathways associated with the core module and the MYC module respectively.
Supplementary Figures
**Figure S1**: Important biological functions related to the 145 computationally predicted human ES cell related TF genes.
**Figure S2**: Important pathways related to the 145 computationally predicted human ES cell related TF genes. Ratio: the ratio of the number of overlapping genes between two gene sets relative to the number of the genes included in the pathway.
**Figure S3**: The network related to the combined gene set with the second highest score is core TFs centered. The combined gene set is made up of the 353 core-bound genes and the 145 computationally predicted human ES cell related TF genes. The core TFs are highlighted in red color.
**Figure S4**: The network related to the combined gene set with the third highest score is TP53-centered. The combined gene set is made up of the 353 core-bound genes and the 145 computationally predicted human ES cell related TF genes.
**Figure S5**: The network related to the MYC target genes with the highest score is MYC-centered.
**Figure S6**: The network related to the MYC target genes with the second highest score is TP53-centered.
**Figure S7**: Top network related to the core module. The three core TFs (in red) form hub nodes in the network.
**Figure S8**: Top network related to the MYC module is MYC-centered.

## Figures and Tables

**Table 1:** Category of the core-bound genes.

	Cytokines and growth factors	Transcription factors	Homeodomain proteins	Cell differentiation markers	Protein kinases	Translocated cancer genes	Oncogenes
Cytokines and growth factors	14						
Transcription factors	0	90					
Homeodomain proteins	0	34	34				
Cell differentiation markers	0	0	0	8			
Protein kinases	0	1	0	3	11		
Translocated cancer genes	0	6	1	1	2	9	
Oncogenes	0	6	1	3	4	9	11

*Some genes are not present in any gene family above.
