# Supplementary figures and images for: Computational Analysis of Transcriptional Circuitries in Human Embryonic Stem Cells Reveals Multiple and Independent Networks
Source: Biomed Res Int. 2014 Jan 9;2014:725780. doi: 10.1155/2014/725780 (PMC3910540; doi:10.1155/2014/725780)

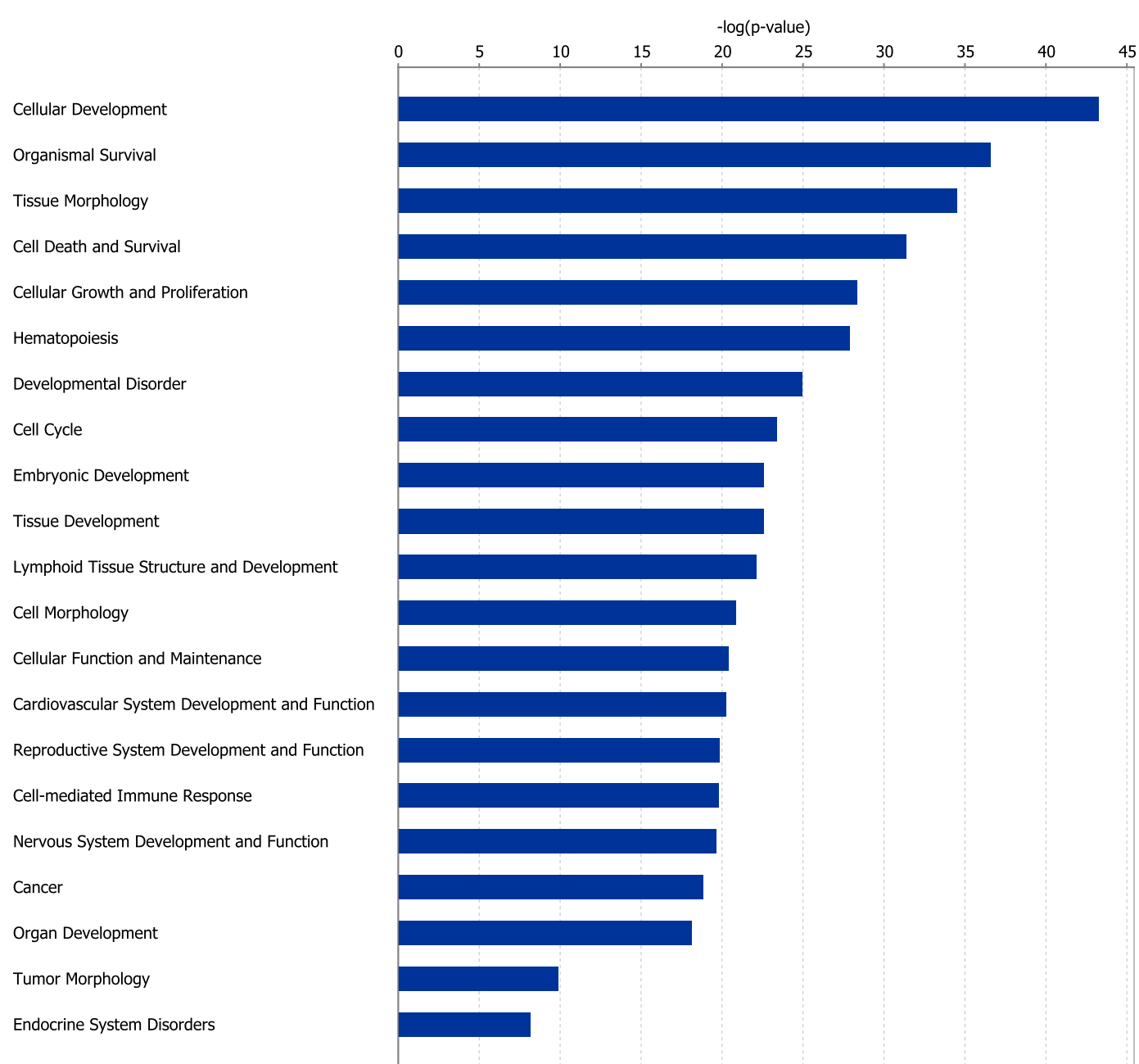

Supplement: Supplementary file 2 [file 725780.f2.zip › supplementary figures_material_checker_2013-12-03_725780_/Figure S1.pdf]

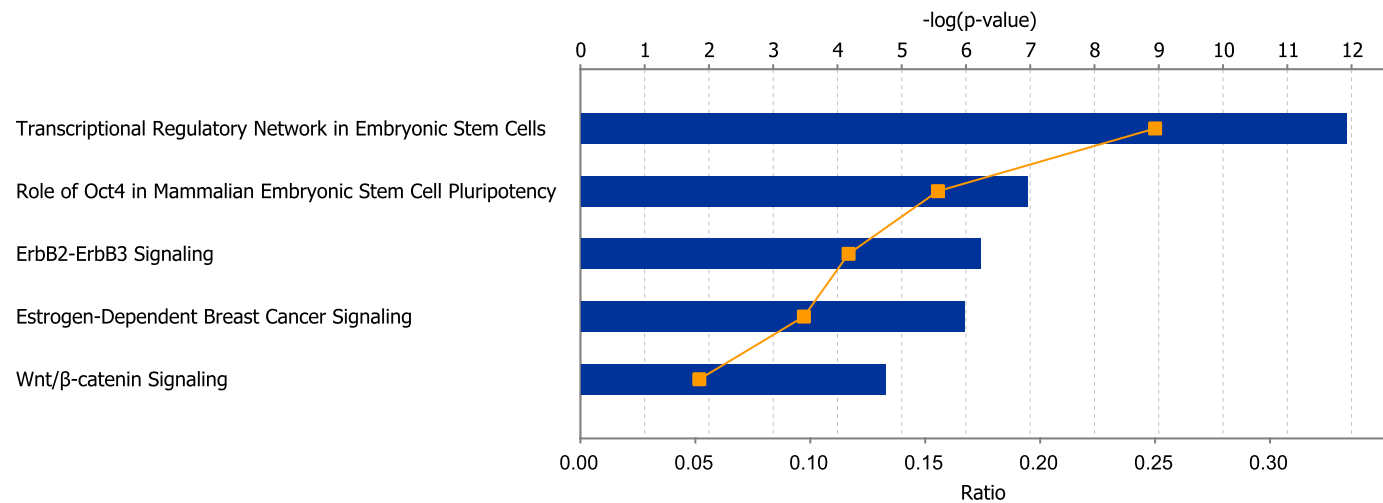

Supplement: Supplementary file 2 [file 725780.f2.zip › supplementary figures_material_checker_2013-12-03_725780_/Figure S2.pdf]

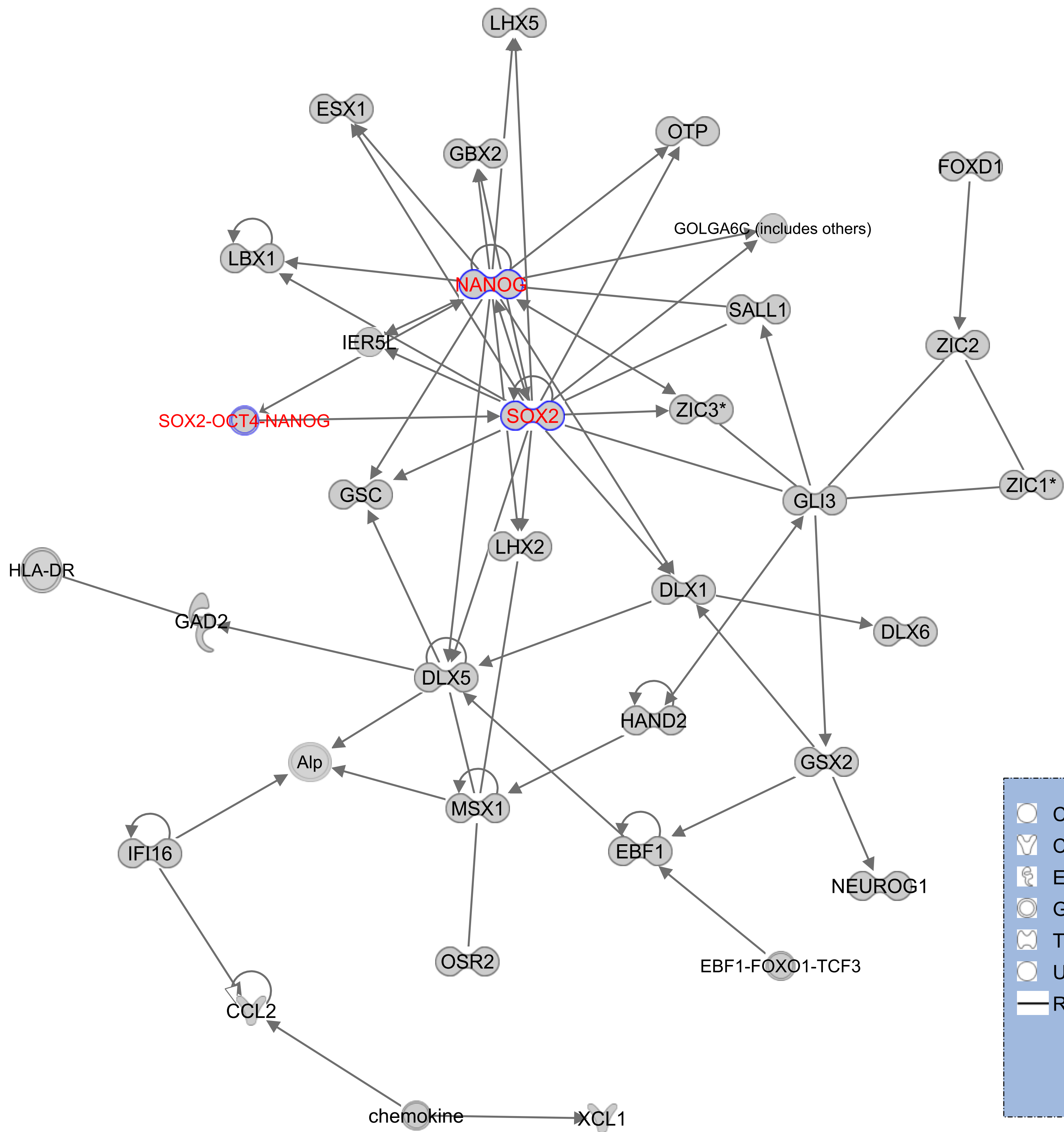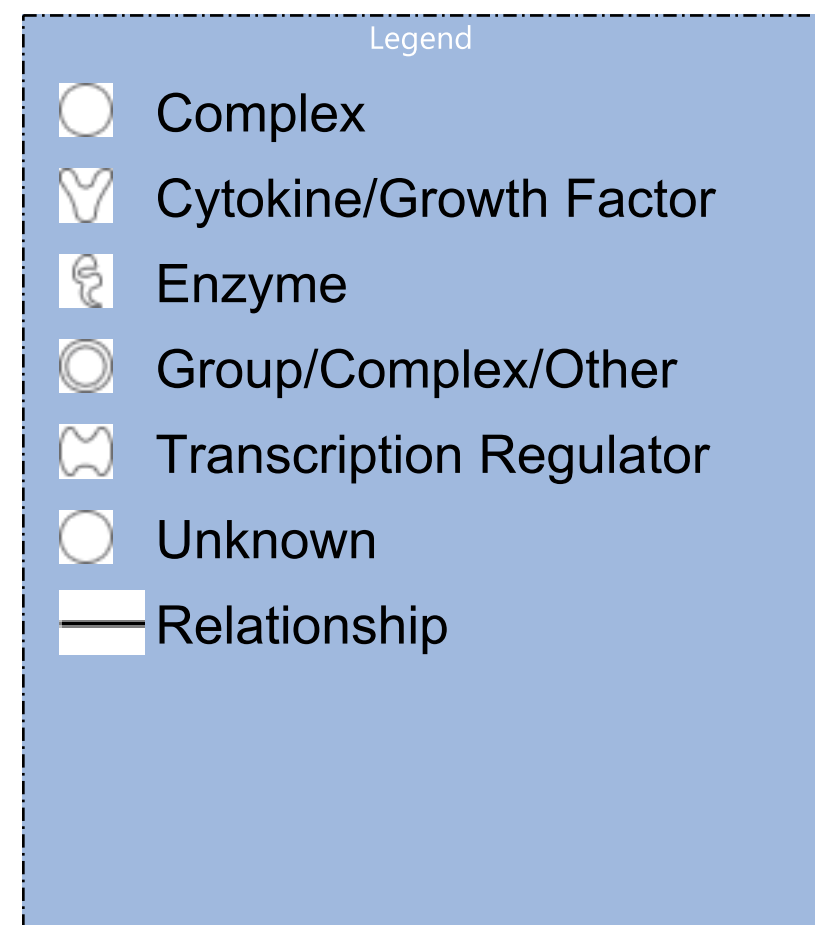

Supplement: Supplementary file 2 [file 725780.f2.zip › supplementary figures_material_checker_2013-12-03_725780_/Figure S3.pdf]

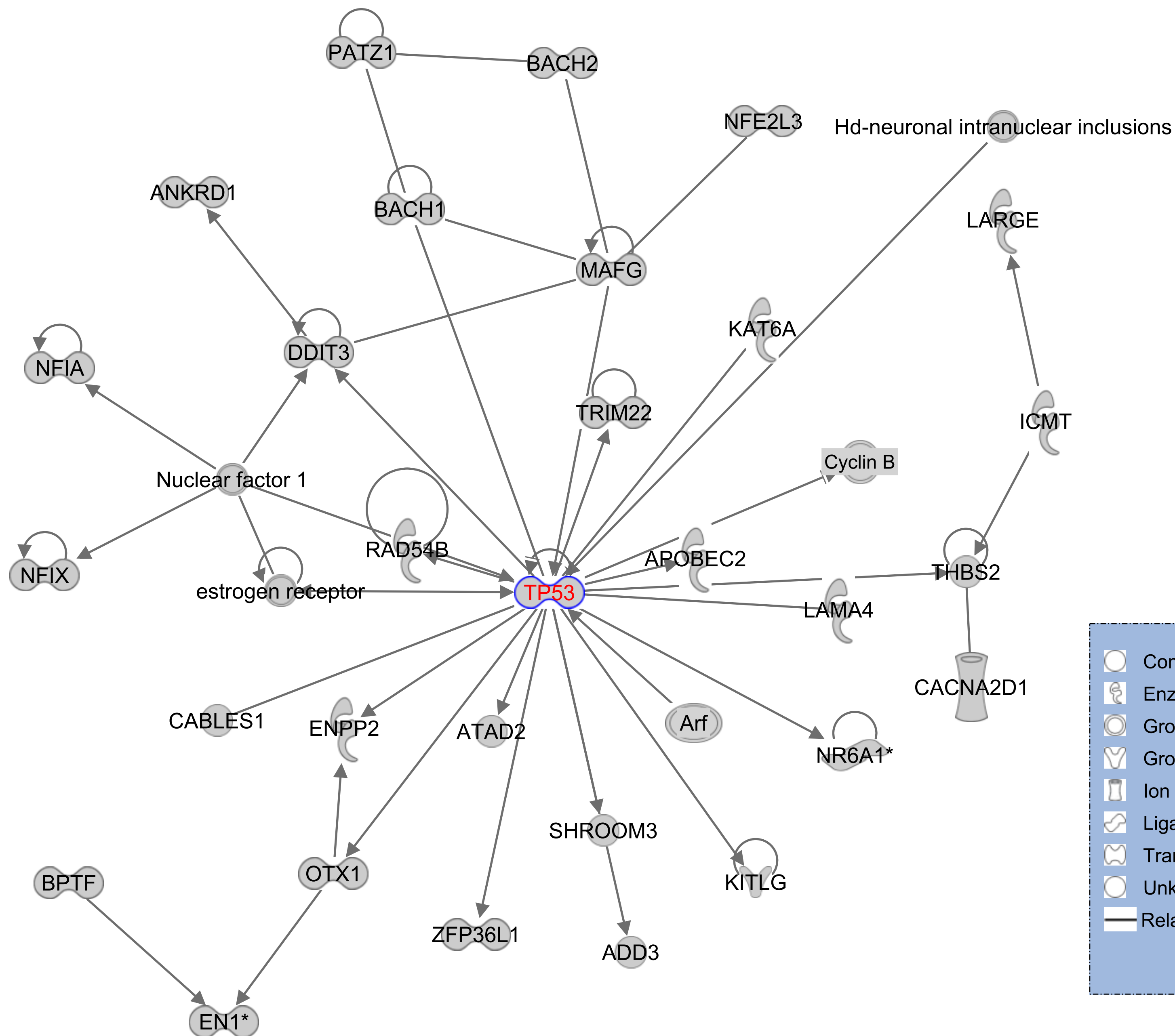

Supplement: Supplementary file 2 [file 725780.f2.zip › supplementary figures_material_checker_2013-12-03_725780_/Figure S4.pdf]

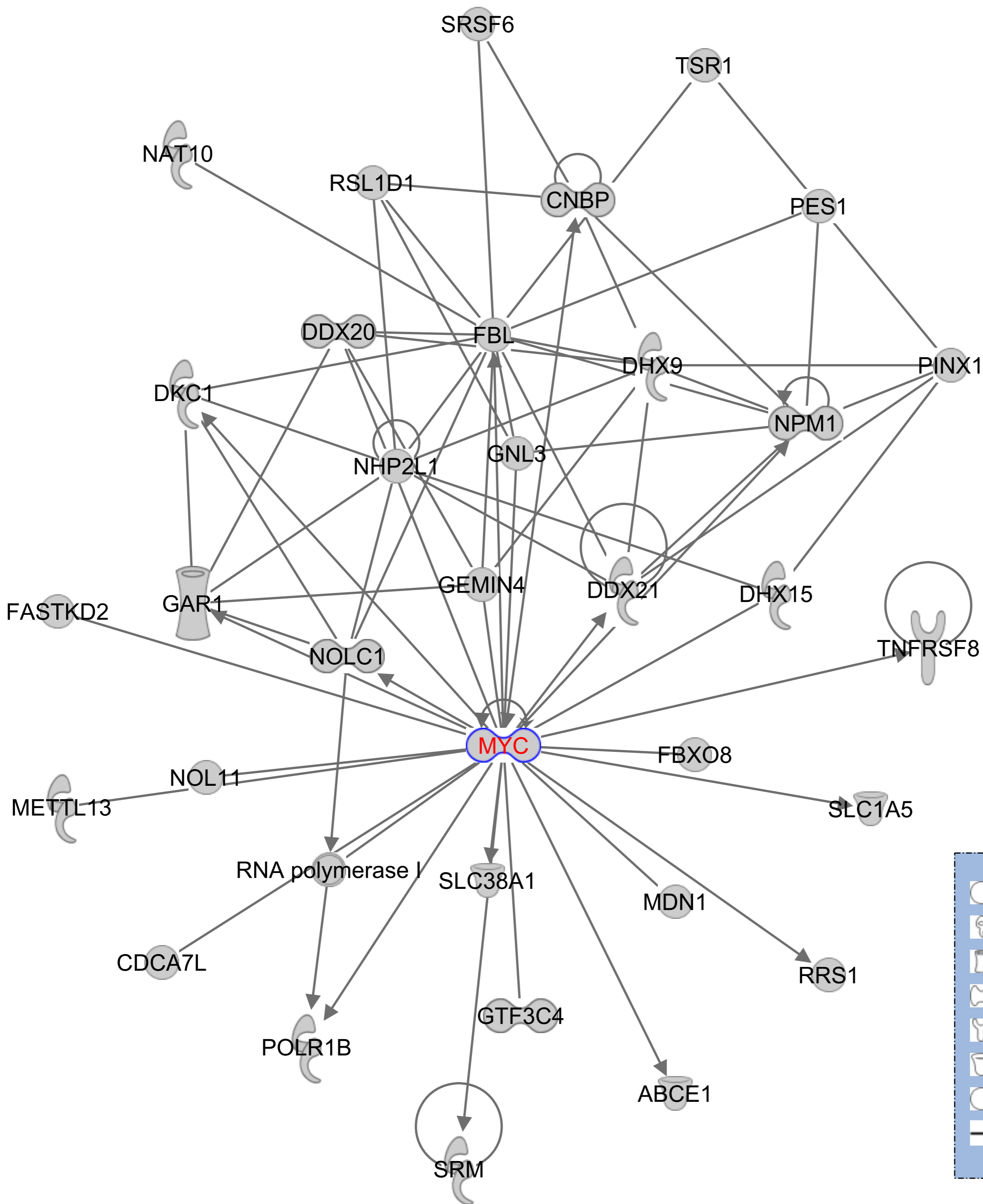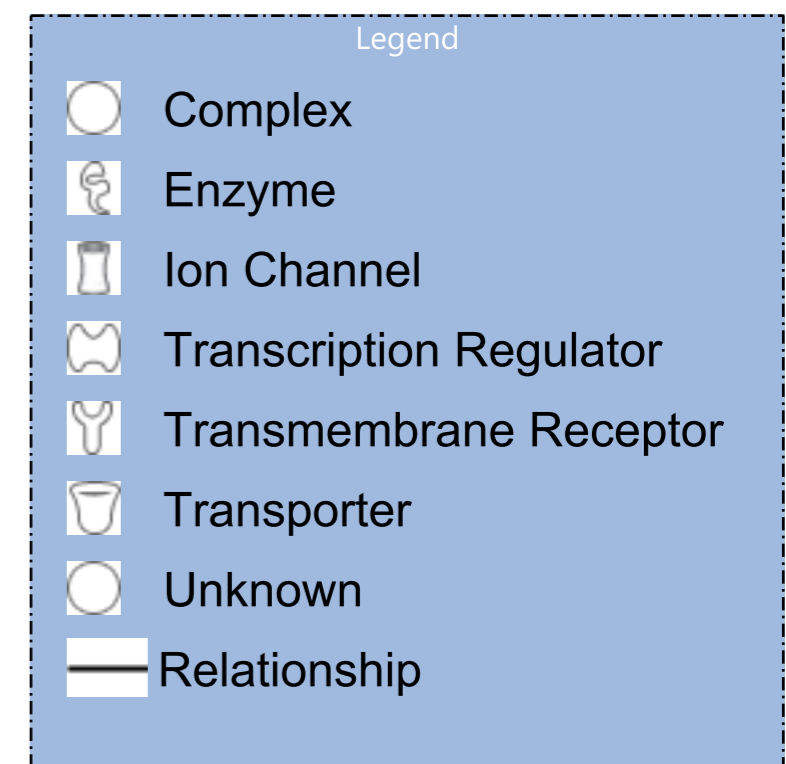

Supplement: Supplementary file 2 [file 725780.f2.zip › supplementary figures_material_checker_2013-12-03_725780_/Figure S5.pdf]

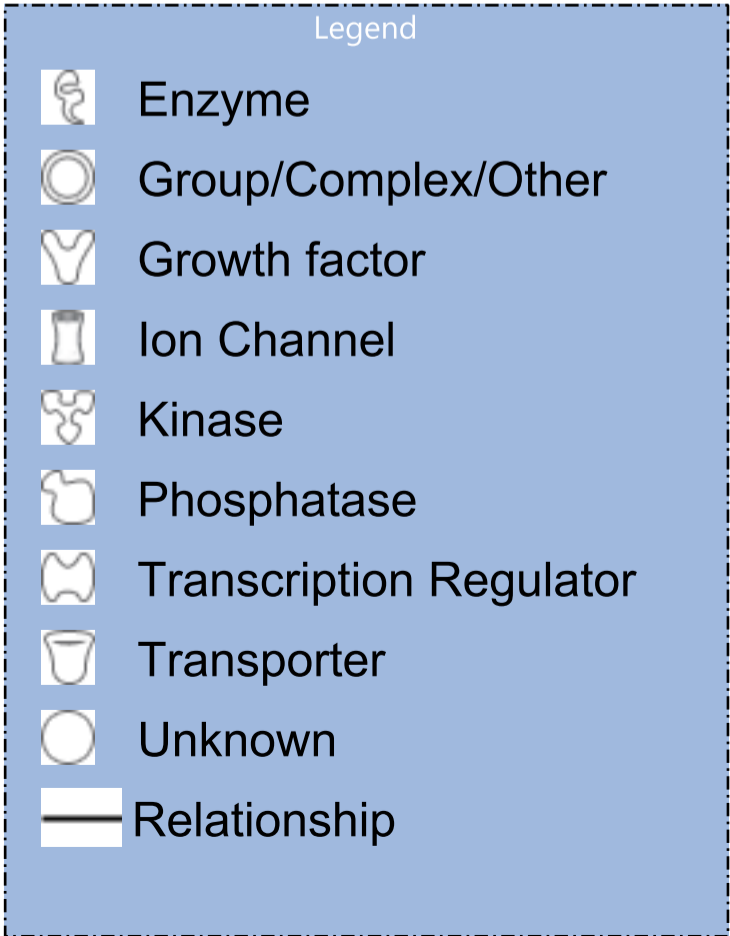

Supplement: Supplementary file 2 [file 725780.f2.zip › supplementary figures_material_checker_2013-12-03_725780_/Figure S6.pdf]

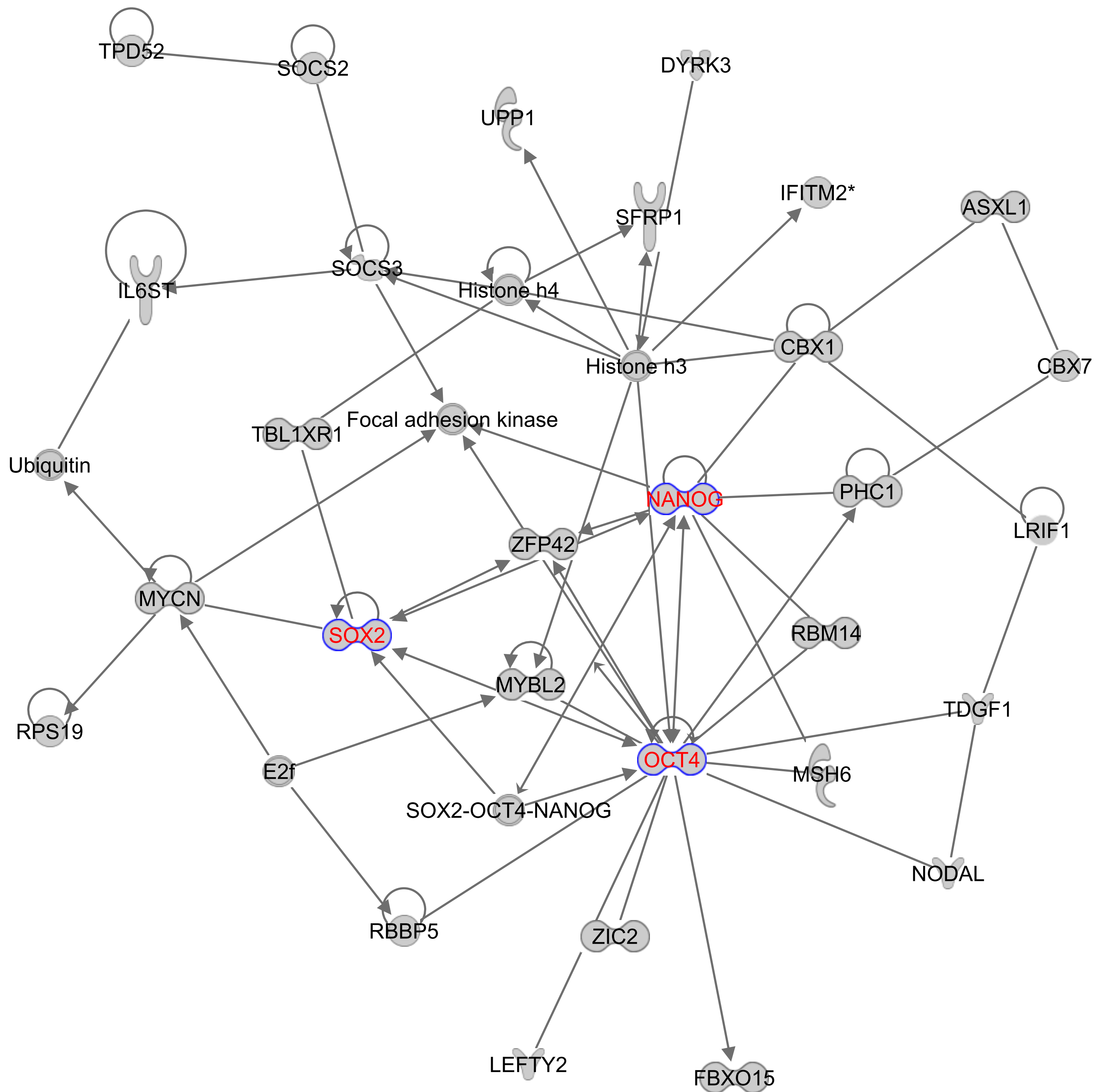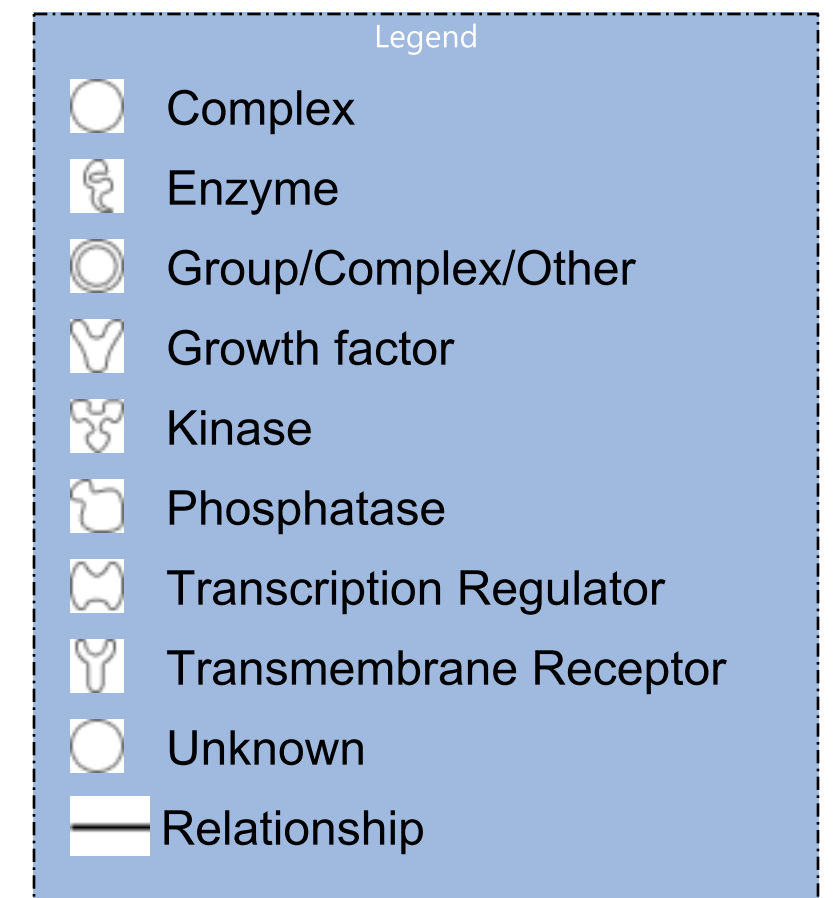

Supplement: Supplementary file 2 [file 725780.f2.zip › supplementary figures_material_checker_2013-12-03_725780_/Figure S7.pdf]

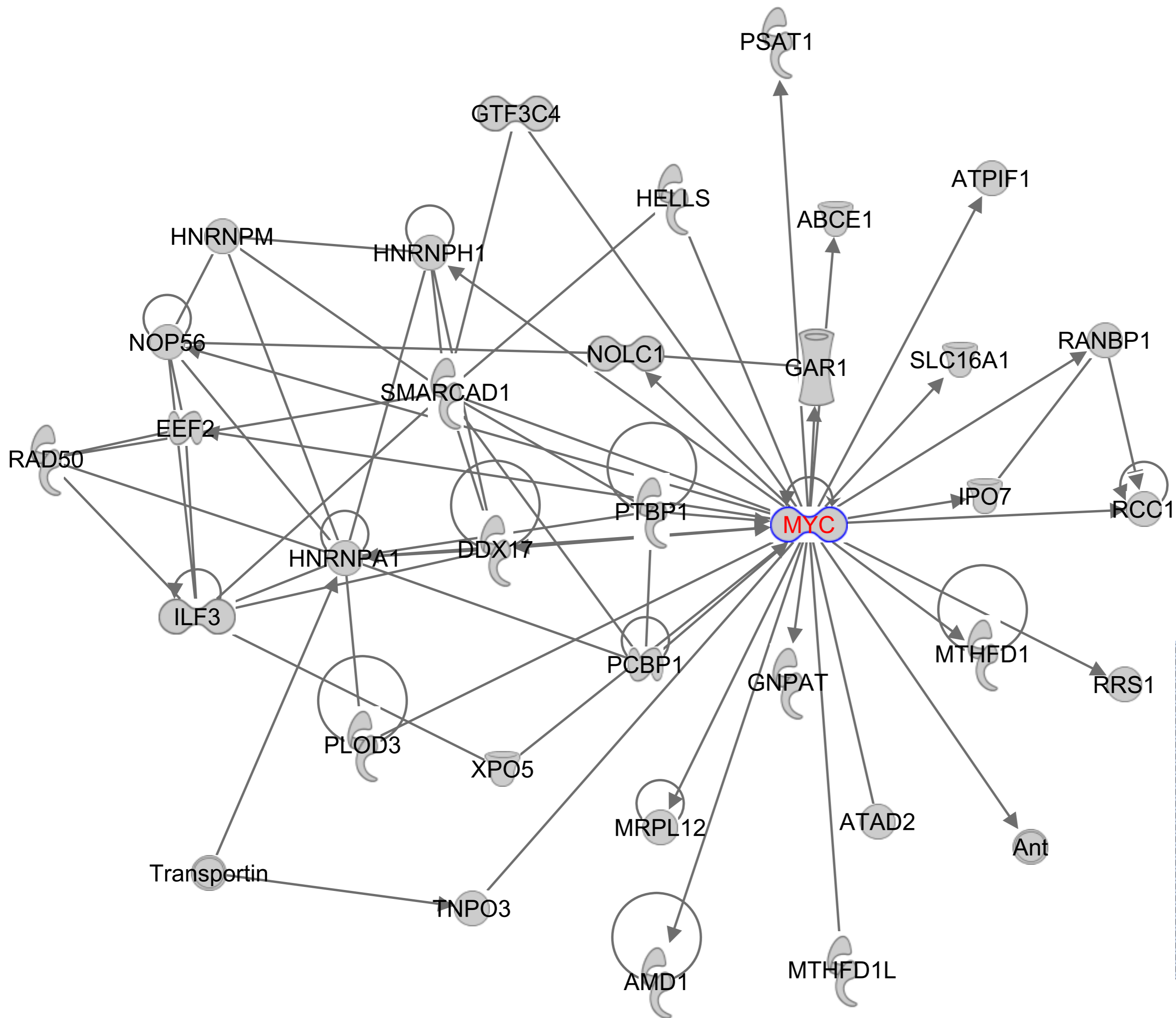

Supplement: Supplementary file 2 [file 725780.f2.zip › supplementary figures_material_checker_2013-12-03_725780_/Figure S8.pdf]
